# Experimental Observations on Perforating Wounds of the Abdominal Viscera

**Published:** 1919-01

**Authors:** 


**_Some Experimental Observations on Perforating Wounds of the Abdominal Viscera._** By HAMILTON DRUMMOND and JOHN FRASER. *Journal Royal Army Medical Corps*, August, 1918.

After a series of experiments on animals the authors found that when single small wounds were made in either small or large intestines the animal invariably recovered.

The next observation showed that several small wounds of the same size could be made in the same loop of intestine and recovery follow. As a rule, when more than five perforations were made in the single loop of bowel, the animal died of general peritonitis.

Certain stomach wounds which, from their anatomical position, are impossible of suture, may close spontaneously,

When small wounds (just sufficiently large to admit number 4 shot) were made, there was a spontaneous recovery.

Wounds of the duodenum and upper jejunum were found to be inevitably fatal.

Wounds of the lower jejunum and ileum showed a strong tendency to spontaneous cure.

Wounds of the large intestine were found to be inevitably fatal.

The authors have made the observation that in perforating wounds of the intestines the omentum does not, primarily at least, come in contact with the injured gut. On the other hand, if the wound has not actually perforated the viscus, but has merely damaged and bruised its wall, the omentum migrates to the injured part and puts itself in such a position that it tends to prevent any further spread of infection.

When bruise-wounds of the intestine are made without actual perforation, the omentum is found to come down to the lesion and to aid in its immediate repair. The same procedure occurs when areas of the gut are devascularized.

In perforating wounds of the gut the omentum behaves differently. If, when the wound is made, the omentum is laid over the injured gut, it remains in this position and therefore takes some share in the subsequent repair.

When the omentum is brought to the site of injury, its greatest benefit will be derived if it is anchored in this position with a few sutures. Apparently the omentum has little control over the intestinal juices of the upper end of the small gut.

The omentum was found capable of replacing a portion of resected stomach wall.

Twenty-four hours after perforating wounds were produced by cutting the bowel wall with fine scissors, there was found to be a glueing together of the intestinal coils in the neighborhood of the lesion.

If the injured bowel at the time of the experiment was covered with omentum, at the end of twenty-four hours the omentum remained extensively glued over the site of the injur.

Examination one week after the original operation showed the omentum adherent only at the points of perforation of the gut. The omentum was firmly adherent to the gut, and a vascular anastomosis could be traced in the shape of fine vessels passing between the omentum and the bowel wall.

On separation of the omentum, the rosette of prolapsed mucous membrane was no longer visible, but the situation of the prolapse was occupied by a plug of yellow fibrin which blocked up each perforation.

During the second and third weeks there was a tendency for sacculation of the mucous membrane to occur at the site of injury. At each point of injury there was a distinct hernia of the mucous membrane through the gap in the muscular wall of the gut into the overlying omentum.

Large areas of stomach wall can be replaced by omental tissue, but restoration of the stomach epithelium does not appear to occur.

